# Shp2 Knockdown and Noonan/LEOPARD Mutant Shp2–Induced Gastrulation Defects

**DOI:** 10.1371/journal.pgen.0030225

**Published:** 2007-12-21

**Authors:** Chris Jopling, Daphne van Geemen, Jeroen den Hertog

**Affiliations:** Hubrecht Institute, Utrecht, The Netherlands; University of Pennsylvania School of Medicine, United States of America

## Abstract

Shp2 is a cytoplasmic protein-tyrosine phosphatase that is essential for normal development. Activating and inactivating mutations have been identified in humans to cause the related Noonan and LEOPARD syndromes, respectively. The cell biological cause of these syndromes remains to be determined. We have used the zebrafish to assess the role of Shp2 in early development. Here, we report that morpholino-mediated knockdown of Shp2 in zebrafish resulted in defects during gastrulation. Cell tracing experiments demonstrated that Shp2 knockdown induced defects in convergence and extension cell movements. In situ hybridization using a panel of markers indicated that cell fate was not affected by Shp2 knock down. The Shp2 knockdown–induced defects were rescued by active Fyn and Yes and by active RhoA. We generated mutants of Shp2 with mutations that were identified in human patients with Noonan or LEOPARD Syndrome and established that Noonan Shp2 was activated and LEOPARD Shp2 lacked catalytic protein-tyrosine phosphatase activity. Expression of Noonan or LEOPARD mutant Shp2 in zebrafish embryos induced convergence and extension cell movement defects without affecting cell fate. Moreover, these embryos displayed craniofacial and cardiac defects, reminiscent of human symptoms. Noonan and LEOPARD mutant Shp2s were not additive nor synergistic, consistent with the mutant Shp2s having activating and inactivating roles in the same signaling pathway. Our results demonstrate that Shp2 is required for normal convergence and extension cell movements during gastrulation and that Src family kinases and RhoA were downstream of Shp2. Expression of Noonan or LEOPARD Shp2 phenocopied the craniofacial and cardiac defects of human patients. The finding that defective Shp2 signaling induced cell movement defects as early as gastrulation may have implications for the monitoring and diagnosis of Noonan and LEOPARD syndrome.

## Author Summary

Shp2 is a protein-tyrosine phosphatase and mutations in Shp2 cause the related Noonan and LEOPARD syndromes in humans. We used the zebrafish to investigate the cell biological role of Shp2 in early development. Shp2 knockdown and expression of mutant Shp2 that contained mutations corresponding to those found in human Noonan and LEOPARD patients, induced similar convergence and extension cell movement defects during gastrulation without affecting cell specification. Active Src family kinases and active RhoA rescued the Shp2 knockdown, indicating that signaling downstream of Shp2 was mediated by Src family kinases and RhoA. Expression of the Noonan and LEOPARD Shp2s in zebrafish induced craniofacial and cardiac defects that were reminiscent of the symptoms observed in human patients. Coinjections demonstrated that Noonan and LEOPARD Shp2s did not cooperate, which is consistent with the two mutants acting in the same signaling pathway with opposing effects. The finding that defective Shp2 signaling induced cell movement defects as early as gastrulation may have important implications for the monitoring and diagnosis of Noonan and LEOPARD syndromes in humans.

## Introduction

Shp2 (*PTPN11*), a nonreceptor protein-tyrosine phosphatase (PTP) with two Src homology 2 (SH2) domains, has a central role in cell signaling. Shp2 is essential for embryonic development and has a role in human disease [1]. Dominant negative Shp2 inhibits mesoderm formation in Xenopus laevis [2]. Gene targeting of Shp2 in Mus musculus leads to truncation of the Shp2 protein resulting in embryonic death around day 8.5, with a range of defects consistent with abnormal gastrulation [3]. Chimeric mice derived from Shp2 ex3^−*/*−^ embryonic stem cells display defective morphogenetic cell movements during gastrulation [4]. Interestingly, bona fide Shp2 null mouse embryos die peri-implantation and Shp2 is required for trophoblast stem cell survival [5].

Activating mutations in Shp2 cause Noonan Syndrome (NS) in humans, whereas LEOPARD syndrome (LS) is caused by dominant negative mutations in Shp2. NS is an autosomal dominant disorder affecting around 1 in 2,000 live births, and is characterized by multiple defects, including short stature, facial abnormalities, and congenital heart defects [6]. Around 50% of NS cases are caused by mutations in Shp2 [7] and 39 different mutations have been identified [8,9]. Most of the NS mutations are localized in the N-SH2 domain or in the PTP domain, and result in activation of Shp2 catalytic activity [10]. The mouse model for NS with an activating D61G mutation bears striking similarities to NS patients, with defects such as short stature, facial dysmorphism, and multiple cardiac defects. Mice homozygous for the mutated gene die prenatally from severe cardiac edema and liver necrosis [11].

LS is an autosomal dominant disease characterized by defects such as lentigines, electrocardiographic defects, ocular hypertelorism, pulmonary stenosis, abnormal genitals, retarded growth leading to short stature, and deafness [12]. Many of the LS symptoms overlap with those seen in NS patients, and LS is also caused by mutations in Shp2. These mutations occur exclusively in the PTP domain of Shp2 disrupting its catalytic activity and leading to dominant negative forms [13,14]. This leads to the conundrum of how mutations in Shp2 with opposing effects on activity induce syndromes in humans with similar symptoms.

Shp2 is known to be involved in many different signaling cascades [15]. Initially, Shp2 was found to have a role in growth factor signaling, downstream of growth factor receptors and upstream of MAP kinase signaling. Zhang et al. [16] demonstrated that Shp2 controls phosphorylation and activation of Src family kinases (SFKs), in that SFK activity is reduced in the absence of Shp2, due to hyperphosphorylation of the inhibitory phosphorylation site. Two SFKs, Fyn and Yes, have a role in vertebrate convergence and extension (CE) cell movements during gastrulation [17]. Moreover, the C-terminal Src kinase, Csk, a negative regulator of SFKs, is crucial for normal gastrulation cell movements as well [18]. Morpholino (MO)-mediated Fyn/Yes knockdown in zebrafish embryos phenocopies Silberblick/Wnt11 and Pipetail/Wnt5 morphants both morphologically and molecularly. Although Fyn and Yes act in a synergistic manner with Wnt5 and Wnt11, they do not function in a linear pathway; instead, they operate in parallel, converging downstream on the small GTPase RhoA. Given the role of Fyn and Yes in gastrulation cell movements, the function of Shp2 as an indirect activator of SFKs, and evidence from *Xenopus* and mouse that Shp2 may have a role in gastrulation cell movements, we hypothesized that Shp2 is involved in CE cell movements during gastrulation.

Here, we show that MO-mediated knockdown of Shp2 in zebrafish embryos resulted in defective CE cell movements during gastrulation, but not cell specification. Genetic epistasis analyses indicated that Shp2 acts through Fyn and Yes, upstream of the small GTPase RhoA. Expression of mutant NS- or LS-Shp2 in zebrafish embryos resulted in overlapping phenotypes, in that normal gastrulation was impaired without affecting cell specification. Specifically, we found that NS-Shp2 induced CE cell movement defects. Moreover, craniofacial and cardiac development were also impaired. The NS- and LS-Shp2s did not act synergistically upon coinjection, which is consistent with the two mutants acting in the same signaling pathway with opposing effects. The defects resulting from expression of mutant Shp2s correspond to symptoms in human NS/LS patients. The notion that defective Shp2 signaling induced cell movement defects as early as gastrulation is important and may have implications for the monitoring and diagnosis of NS and LS.

## Results/Discussion

### Shp2 Knockdown Induced Defects in CE during Gastrulation

**Figure 1:**
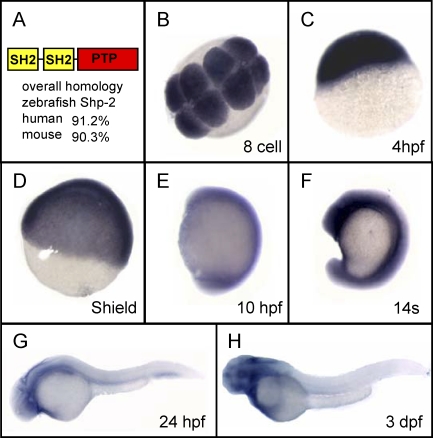
Zebrafish Shp2 Is Conserved and Is Expressed Ubiquitously during Development (A) Schematic representation of zebrafish Shp2 with two SH2 domains to the N-terminal side of the PTP domain. The overall sequence identity with human and mouse Shp2 is indicated. (B–F) In situ hybridization with a Shp2-specific antisense probe at various stages of development: (B) 8-cell stage, (C) 4 hpf, (D) shield stage, (E) 10 hpf, (F) 14 somite (14 s, 14 hpf), (G) 24 hpf, and (H) 3 dpf.

**Figure 2:**
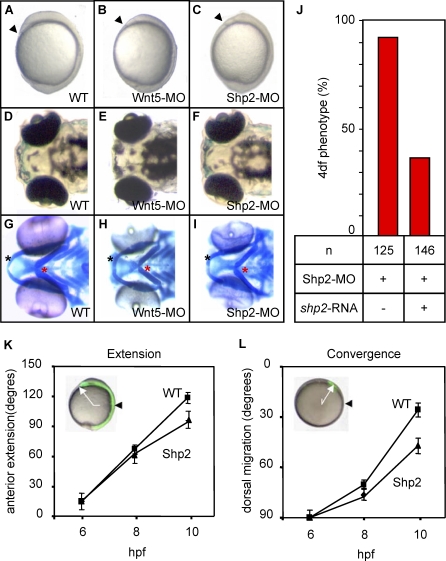
Shp2-MO–Induced CE Cell Movement Defects Zebrafish embryos were not injected (A, D, G) or microinjected with Wnt5-MO (5 ng) (B, E, H) or Shp2-MO (1.0 ng) (C, F, I) at the one-cell stage. (A–C) Morphology at 10 hpf shows reduced anterior extension of the Wnt5-MO– and Shp2-MO–injected embryos. Arrowheads indicate the anterior of the embryos (D–F) Morphology of the Wnt5 and Shp2 knockdown embryos at 4 dpf show a mild hammerhead-like phenotype. (G–I) Alcian blue staining of the cartilage in the heads of 4 dpf embryos. Black asterisk, Meckel's cartilage; red asterisk, ceratohyal. (J) Zebrafish embryos were (co-) injected with Shp2-MO (1.0 ng) and 300 pg human *shp2* mRNA and scored at 4 dpf. (K,L) Embryos were loaded with caged fluorescein dextran and the fluorophore was uncaged at the shield stage (6 hpf) dorsally to determine anterior extension (K, white arrow in inset; site of uncaging, black arrowhead) or laterally to determine dorsal migration (L, white arrow in inset; initial position at the shield stage, black arrowhead). Cell labeling of the same embryos was followed immediately after uncaging at 80% epiboly (8 hpf) and at tailbud stage (10–10.5 hpf). WT and Shp2-MO–injected embryos were assessed and averages for ten embryos are given in degrees.

Zebrafish Shp2 (Ensembl database ENSDARG00000020334, http://www.ensembl.org) is highly homologous to human and mouse Shp2 (91.2% and 90.3% protein sequence identity, respectively) (Figure 1A). In situ hybridization experiments show that *shp2* is ubiquitously expressed during early zebrafish development (Figure 1B–1F). At 24 h post fertilization (hpf) and 3 d post fertilization (dpf), *shp2* was broadly expressed, with enhanced levels of expression in the anterior parts of the embryo (Figure 1G and 1H). We designed a Shp2-MO targeting the start codon and injected it at the one-cell stage. We found that 1ng Shp2-MO consistently produced specific defects in embryonic development. The first visible defect is a failure of the embryo to extend properly around the yolk at 10 hpf (Figure 2A–2C). At later stages (4 dpf), embryos were shorter and developed a hammerhead phenotype similar to Wnt5 morphants (Figure 2D–2F). Alcian blue staining of 4 dpf embryos indicated that the cartilaginous structures in the head of Shp2 and Wnt5 morphants reside more posteriorly than in uninjected controls; compare Meckel's cartilage (black asterisk) and the ceratohyal (red asterisk) (Figure 2G–2I). We quantified the hammerhead phenotype by assessment of the ratio of the distance to the tip of the nose divided by the distance between the eyes (as illustrated in Figure S1). There was a significant difference in this ratio between wild-type (WT) (1.87 ± 0.03, *n* = 4) and Shp2-MO injected (1.40 ± 0.20, *n* = 10) embryos (*p* < 0.001, student's *t*-test), demonstrating that there was a significant craniofacial defect following Shp2 knockdown. Coinjection of 300 pg human *shp2* RNA rescued all Shp2-MO-induced defects (Figure 2J). Higher amounts of Shp2-MO induced more severe defects (unpublished data), but not all defects were rescued by coinjection of synthetic RNA. Therefore, we used 1 ng Shp2-MO for all subsequent knockdowns.

Reduced extension of the body axis upon Shp2 knockdown is consistent with CE cell movement defects. Therefore, we directly investigated whether Shp2 knockdown affected CE cell movements by cell tracing. Caged fluorescein was (co-) injected at the one-cell stage. At 6 hpf, a cluster of cells within the dorsal shield was labeled by uncaging the caged fluorescein with a short, localized pulse of UV light. This group of cells was monitored every 2 h during gastrulation. The distance the cells migrated is directly proportional to embryonic extension. Repeating the process at 90° to the shield gives an effective measurement of convergence of the mesendodermal cells. Shp2 knockdown resulted in a significant reduction in CE during gastrulation (Figure 2K,L). These results demonstrate that Shp2 knockdown induced defects in CE cell movements during gastrulation.

### Shp2 Knockdown Did Not Affect Cell Specification

**Figure 3:**
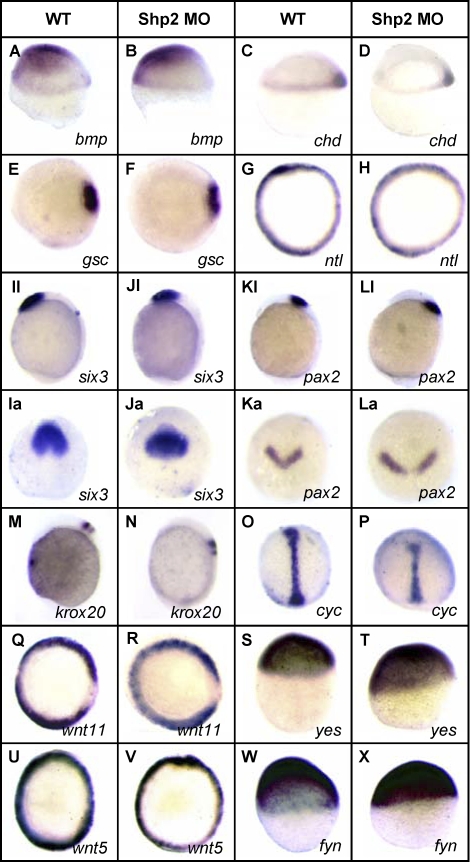
Shp2 Knockdown Did Not Affect Cell Specification Control and Shp2-MO–injected embryos were fixed at 6 hpf (A–H, Q–X), 8 hpf (O,P), or 10 hpf (I–L), and in situ hybridization was done with the indicated probes. Either lateral views (A–D, Il–Ll, M, N, S, T, W, X) or animal pole views (E–H, Ia-La, O–R, U, V) are depicted here.

Shp2 is known to be involved in many different signaling cascades [15]. In fact, in a recent study in *Drosophila*, over 40 different genes associated with at least four separate pathways (EGFR, Notch, DPP, and Jak/Stat) were found to interact with gain-of-function Shp2 mutants [19]. Some of these pathways are important for proper cell specification and defective signaling leads to a variety of phenotypes, including defects in the A-P axis. We used a panel of well-characterized in-situ markers that have been used before to assess effects on cell specification [20–23]. *Bone morphogenetic protein 2b* (*bmp2b*) is involved in the specification of ventral cell fates but was not affected by knockdown of Shp2 (Figure 3A and 3B). Expression of the dorsalizing factor *chordin* (*chd*) remained the same (Figure 3C and 3D), as did the dorsal specific gene *goosecoid* (*gsc*), which is expressed in the organizer (Figure 3E and 3F). Expression of the mesendodermal marker *notail* (*ntl*) also remained unchanged in Shp2 morphants (Figure 3G and 3H). These markers indicate that knockdown of Shp2 did not alter cell fate in early zebrafish embryos, suggesting it has a role in CE cell movements, rather than cell specification.

Expression of the anterior brain markers, *six3* (forebrain), *pax2* (mid-hindbrain boundary), and *krox20* (rhombomeres 3 and 5) persisted in the Shp2 morphants, indicating that the structures these markers delineate were present. However, the expression patterns of these three genes were shifted posteriorly (Figure 3Il–Ll), which—in the case of *pax2* and *six3*—was accompanied by a broader expression pattern (Figure 3Ka–La). At 8 hpf, *cyclops* (*cyc*) is expressed in axial mesendodermal cells of gastrulating embryos. Shp2 morphants clearly have a shorter expression pattern when compared to uninjected controls (Figure 3O and 3P). These results are consistent with disrupted convergence and extension cell movements upon knockdown of Shp2.

Shp2 might regulate CE cell movements by modulation of expression of the noncanonical Wnts, or by Fyn and Yes, known regulators of CE cell movements. However, the expression of *wnt11*, *wnt5*, *fyn*, and *yes* remained unaffected in Shp2-MO injected embryos (Figure 3Q–3X), indicating that Shp2 has a more direct role in CE signaling.

### Shp2 Acts through Fyn,Yes, and RhoA in Gastrulation Cell Movements

In order to assess the mechanism underlying Shp2-mediated cell movements in gastrulation, we coinjected MOs with synthetic mRNAs and assessed CE cell movement defects by measurement of the angle between the most anterior and the most posterior tissues at 10 hpf. The Shp2-MO by itself induced an increase in the angle between the extremes of the developing embryo, which was rescued by coinjection of synthetic human *shp2* mRNA (Table 1). Coinjection of RNA encoding constitutively active Fyn and Yes with Shp2-MO also rescued the Shp2 morphants (Table 1), indicating that Fyn and Yes are genetically downstream of Shp2. Fyn- and Yes-MOs induced severe reductions in embryo body axis extension, and coinjection with synthetic *shp2* mRNA did not rescue (Table 1), confirming that Shp2 is upstream of Fyn and Yes. Low amounts of Shp2-MO together with Wnt5-MO, which did not induce defects when coinjected alone, induced a hammerhead phenotype at 4 dpf (Figure S2), indicating that Shp2 and Wnt5 interact genetically. However, *Wnt5* mRNA did not rescue the Shp2 morphants and *shp2* mRNA did not rescue Wnt5 morphants (Table 1), indicating that Shp2 and Wnt5 do not operate in the same linear genetic pathway. Active RhoA rescued the Shp2 morphants (Table 1), which is consistent with Shp2 being upstream of Fyn and Yes, which, in turn, act upstream of RhoA.

**Table 1:**
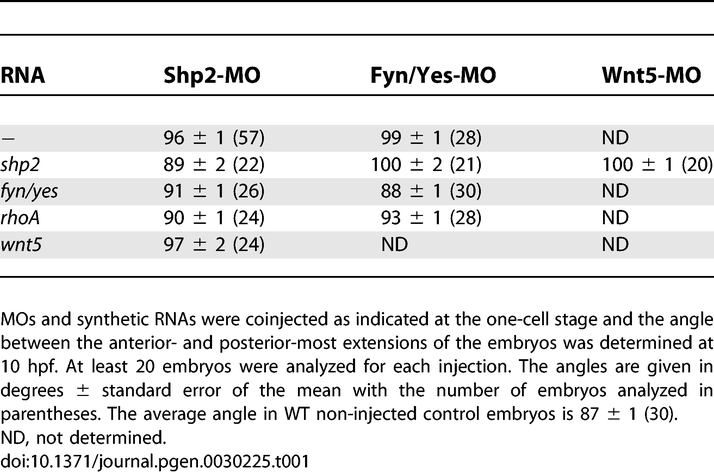
Shp2 and Fyn/Yes Signal in Parallel to Wnt5, Converging on RhoA

Shp2 is most commonly associated with the Ras/MAPK pathway, which regulates many developmental processes, such as cell proliferation and cell specification [24,25]. However, the phenotype we observed in the Shp2 knockdown embryos was not consistent with a massive reduction in cell proliferation, nor with changes in cell specification (Figure 3). Given that the Shp2 knockdown phenotype was rescued by coinjection of active RhoA, the Ras/MAPK signaling pathway appeared not to be essential for Shp2 signaling during gastrulation. Instead, we implicate SFKs and RhoA downstream in the Shp2 signaling cascade. In Xenopus laevis, mutant, active Shp2 induces elongation of animal cap explants, which is blocked by coexpression of dominant negative RhoA [26], suggesting involvement of RhoA rather than Ras/MAPK, similar to what we observed in early zebrafish embryos. In a recent report, Shp2 knockdown was reported to induce craniofacial hypoplasia and heart malformations, similar to Raf1 knockdown [27]. Mutations in Raf1 were linked to NS [27,28]. Other Ras/MAPK signaling components were identified in NS as well, including KRAS and SOS1 [29–31]. Although we cannot exclude that Ras/MAPK signaling has a role in Shp2 signaling in gastrulation cell movements, we demonstrate here that we can rescue the Shp2 knockdown phenotype with active SFKs or active RhoA, indicating that SFKs and RhoA are downstream of Shp2 in gastrulation cell movements.

### Noonan and LEOPARD Syndrome Shp2-Induced CE Cell Movement Defects

**Figure 4:**
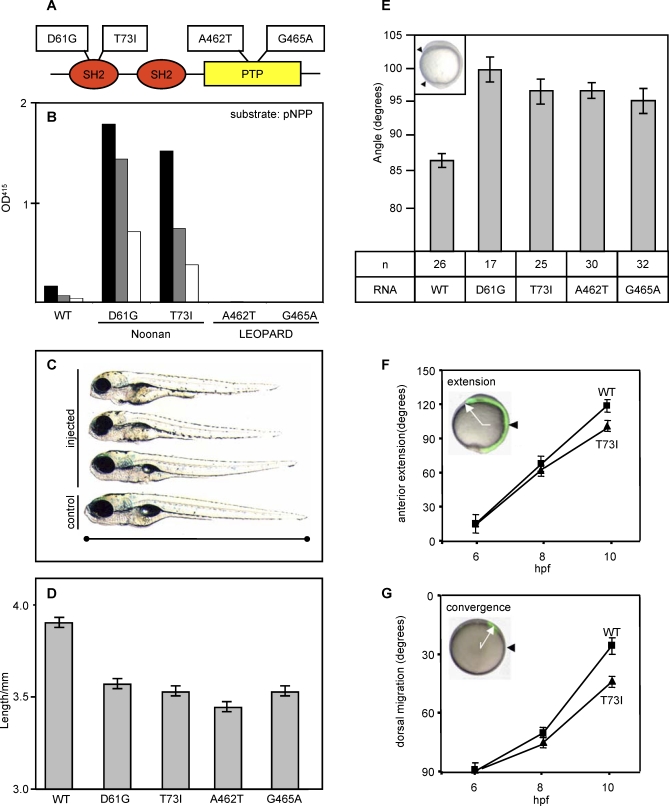
NS and LS Mutant Shp2 Expression Induced CE Cell Movement Defects during Gastrulation (A) Schematic representation of zebrafish Shp2 with the NS and LS mutations indicated. (B) PTP activity of WT Shp2, NS (D61G and T73I) and LS (A462T and G465A) mutant Shp2 was assayed using *p*-nitrophenylphosphate and quantified spectrophotometrically. Each experiment was done with three different amounts of GST-fusion protein (black bar, high; grey bar, middle; and white bar, low). (C) NS- and LS-Shp2 expression reduced zebrafish embryo body length. Three injected embryos (D61G, 150pg) at 4 dpf are depicted with a noninjected control embryo at the bottom. The figure is representative of defects associated with all NS- and LS-Shp2 expressing embryos. (D) The length of the embryos was measured at 4 dpf and the average is shown here. Two tailed student *t*-tests indicate a significant decrease in length after injection of each NS or LS RNA ( *p* < 0.001). (E) NS- and LS-Shp2 RNA injection results in reduced extension of the body axis at 10 hpf. The angle between the most anterior and posterior embryonic structure (arrowheads in inset) was determined at the one-somite stage and the average angle is depicted here in degrees. Two tailed student *t*-tests indicate a significant increase in the angle after injection of NS or LS RNA (*p* < 0.001). The number of embryos used here is indicated (n). (F, G) Cell tracing was done as described in Figure 2. NS-Shp2 (T73I) was coinjected with caged fluorophore at the one-cell stage and after uncaging at 6 hpf, extension and convergence were determined at 8 hpf and 10 hpf in ten embryos per condition.

To investigate the use of zebrafish as a model for NS and LS, we generated two NS-Shp2 and two LS-Shp2 mutants by introducing mutations into zebrafish Shp2, as found in NS and LS patients, respectively (Figure 4A). For NS, we substituted Asp61 with Gly (D61G) or Thr73 with Ile (T73I). For LS, Ala462 was mutated to Thr (A462T), or Gly465 to Ala (G465A). The two NS proteins showed a 6-fold increase in activity compared to WT Shp2 in in vitro PTP assays, whereas the two LS-Shp2s did not exhibit detectable PTP activity (Figure 4B). These results are consistent with catalytic activity data of mammalian NS and LS Shp2 mutants [10,14].

To determine how mutant Shp2 affects the development of zebrafish, we injected synthetic RNA encoding NS-Shp2 or LS-Shp2 into embryos at the one-cell stage. We titrated the amount of RNA down to amounts that reproducibly induced specific phenotypes (D61G, 150pg; T73I, 100pg; A462T, 75pg; and G465A, 50pg). These phenotypes were not observed in embryos injected with green fluorescent protein (GFP) RNA (300pg). Similar amounts of RNA encoding WT Shp2 (150–300 pg) did not induce defects. Very high amounts of WT Shp2 RNA (>800 pg) induced phenotypes, similar to NS- and LS-Shp2, albeit the phenotypes were not as severe (unpublished data), indicating that NS- and LS-Shp2 had strong, dominant functions. Injection of NS- or LS-Shp2s resulted in significantly shorter embryos at 4 dpf when compared to noninjected or GFP-injected controls (Figure 4C and 4D). Body axis extension was already reduced at 10 hpf as the angle between the most anterior and posterior tissues was significantly increased upon injection of each of the NS- and LS-Shp2s (Figure 4E). Cell tracing experiments demonstrated that both extension (Figure 4F) and convergence (Figure 4G) were reduced significantly upon injection of mutant T73I NS-Shp2. In situ hybridization with *ntl* and *gsc* markers on NS- and LS-injected embryos demonstrated that cell specification was not affected (Figure S3). These results demonstrate that expression of NS-Shp2 induced defective CE cell movements during gastrulation without affecting cell specification.

### NS- and LS-Shp2–Induced Defects Resembled Symptoms in NS and LS Patients

**Figure 5:**
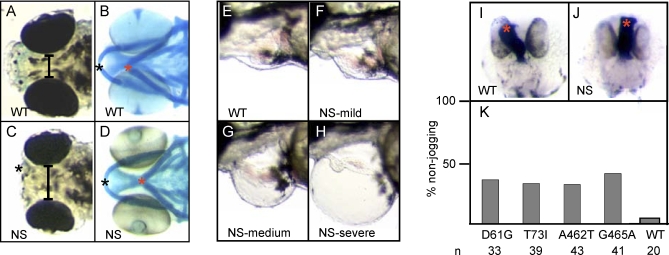
Craniofacial and Heart Defects upon NS or LS RNA Injection (A,C) Extension of anterior structures (black asterisk) was impaired and the eyes were spaced wider apart (black bar) in NS-Shp2–injected embryos (D61G, 150 pg) (C) than in the noninjected control (A). The figure is representative of defects associated with all NS- and LS-Shp2–injected embryos. (B,D) Alcian blue staining of the cartilage in the head of NS-Shp2 injected embryos (D) compared to noninjected control (B). Black asterisk, Meckel's cartilage; red asterisk, ceratohyal. (E–K) NS- and LS-induced heart defects. (E–H) Morphology of representative 3 dpf embryos with (E) noninjected control, (F) mild, (G) intermediate, and (H) severe heart defect. Phenotypes evoked by NS- and LS-Shp2 were indistinguishable. (I–K) *cmlc2* in situ hybridization marks the heart. (I) Noninjected control with normal heart jogging (red asterisk). (J) Injected embryo showing defective cardiac jogging (red asterisk). (K) Quantification of heart jogging of NS- or LS-Shp2 RNA–injected embryos stained with *cmlc2*, depicted as percent non-jogging.

Embryos injected with either NS- or LS-Shp2 RNA developed craniofacial abnormalities that were apparent at 4 dpf. Notably the eyes were set wider apart and anterior structures had not extended normally (Figure 5A–5D). Alcian blue stainings of cartilaginous structures showed that structures, including Meckel's cartilage (black asterisk) and the ceratohyal (red asterisk), resided more posteriorly than in WT controls. Failure of anterior structures to extend normally and wider spacing of the eyes in NS- or LS-Shp2 expressing zebrafish embryos was similar to the Shp2 knockdown zebrafish embryos (Figure 2). Moreover, the facial abnormalities in NS- and LS-Shp2 expressing zebrafish were reminiscent of the symptoms that are observed in NS/LS patients and the NS mouse model. There is no evidence to suggest that this phenotype was caused by defective gastrulation cell movements. Mutants with disrupted gastrulation such as *wnt5* [32] and *knypek* [33] develop similar craniofacial anomalies. Rescue of the *knypek* mutant by RNA injection led to rescue of the gastrulation defects, but not of the craniofacial defects, indicating that these defects are independent [33].

Injection of NS- or LS-Shp2 RNA caused defects in heart development. Similar defects were observed upon injection of NS- or LS-Shp2, and the defects varied in penetrance from mild to grossly edematous, as illustrated for NS-Shp2 (Figure 5E–5H). In situ hybridization using the heart-specific probe *cmlc2* at 24 hpf demonstrates that the heart of NS/LS-injected embryos failed to jog to the left in approximately 30% of the NS- or LS-Shp2–injected embryos (Figure 5I–5K). Homozygous NS mutant mice develop a grossly edematous heart [11], similar to NS and LS zebrafish, indicating that the injected zebrafish phenocopy the symptoms observed in human patients and in gene-targeted mice.

The CE cell movement defects that we observed are most likely resulting from defective directional cell movements, which in turn result from impaired cell polarization. We hypothesize that the craniofacial and cardiac defects we observed may also result from defective cell movements of neural crest cells shaping the face and myocardial cells shaping the heart, respectively. Future work should focus on the role of Shp2 in polarization and migration of these cells.

### NS- and LS-Shp2 Do Not Cooperate

**Figure 6:**
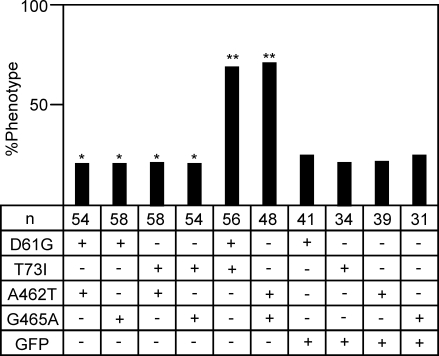
NS and LS Do Not Cooperate Embryos were coinjected with combinations of suboptimal concentrations of NS-Shp2 (T73I, 75pg or D61G, 100pg), LS-Shp2 (A462T, 50pg or G465A, 30pg), or GFP RNA (150pg). Embryos were assessed at 4 dpf and scored for any of the previously documented phenotypes. Chi^2^ tests indicate a significant increase in the observed phenotype after coinjection of the two NS-Shp2s or the two LS-Shp2s compared to single NS- or LS-Shp2s with GFP (*p* < 0.001, double asterisks). Combinations of NS- and LS-Shp2s (single asterisk) are not significantly different from NS- or LS-Shp2 with GFP. The number of embryos that were analyzed is indicated (n).

Finally, we investigated how activating and inactivating mutations in Shp2 might produce similar phenotypes in early zebrafish development. To this end, we coinjected suboptimal amounts of combinations of either NS-Shp2, LS-Shp2, or GFP, which did not induce phenotypes by themselves, and embryos were assessed at 4 dpf. Coinjection of the two NS-Shp2s (D61G and T73I) resulted in a significant increase in the observed phenotypes compared to coinjection of D61G or T73I with GFP (Figure 6). Likewise, coinjection of the two LS-Shp2s (A462T and G465A) induced an increase in phenotypes as compared to control A462T or G465A coinjections with GFP. In contrast, coinjection of suboptimal amounts of combinations of NS-Shp2 RNA with LS-Shp2 RNA did not lead to a significant increase in the number of affected embryos (Figure 6). These results demonstrate that combinations of NS-Shp2 and LS-Shp2 do not act synergistically, whereas suboptimal amounts of NS-Shp2 mutants or LS-Shp2 mutants cooperate to induce defects in zebrafish embryos.

The observation that activation and inhibition of the same signaling pathway induces similar phenotypes is not unprecedented. Microinjection of RNA encoding Rok2 or Galpha12/13 induces similar gastrulation defects as knockdown of Rok2 or Galpha12/13, respectively [21,22]. An activity window exists for these factors. If overall activity falls outside of this window, the resulting phenotypes are very similar. We established that coinjection of two different NS-Shp2s or LS-Shp2s led to an increase in the number of affected embryos. Our results are consistent with NS-Shp2 and LS-Shp2 acting in the same pathway, with one activating and the other inhibiting signaling.

### Conclusion

We show here that knockdown of Shp2 induced CE cell movement defects, but did not affect cell specification. These defects were rescued by active Fyn and Yes and active RhoA, indicating that SFK-Rho signaling, rather than MAPK signaling was required for this function of Shp2. Expression of NS- or LS-mutant Shp2 RNA in zebrafish embryos led to overlapping phenotypes, characterized by craniofacial and cardiac defects, reminiscent of the symptoms observed in NS/LS patients. The notion that defective Shp2 signaling induced cell movement defects as early as gastrulation is important and may have implications for the monitoring and diagnosis of NS and LS.

## Materials and Methods

### Zebrafish and in situ hybridization.

Zebrafish were kept and the embryos were staged as described before [34]. In situ hybridizations were done essentially as described [35] using probes specific for *bmp2b* [36], *chd* [37], *cmcl2* [38], *cyc* [39], *gsc* [40], *krox20* [41], *ntl* [42], *pax2* [43], *six3* [44], *wnt5* [45], *wnt11*, *fyn*, and *yes* [18].

### Constructs.

The mutations D61G, T73I, A462T and G465A were introduced into zebrafish Shp2 by site-directed mutagenesis and cloned into EcoR1/BamH1 sites of pBSK11 and verified by sequencing. Fusion proteins were expressed from pGEX-based bacterial vectors encoding GST fusion proteins of WT Shp2 and all four NS/LS mutated constructs. Fusion proteins were produced in bacteria and purified using standard procedures.

### MOs, RNA, and injections.

Antisense MOs were designed to include the start ATG of the respective cDNAs and ordered from GeneTools (Philomath): Shp2, 5′-GGTGGAACCACCTTCGGGATGTCAT. The Fyn, Yes, and Wnt5 MOs were described previously [17]. 5′ capped sense RNAs were synthesized using the mMessage mMachine kit (Ambion). The amount of RNA that was injected at the one-cell stage was optimized for each synthetic RNA. For the rescue experiments, we used mutant, constitutively active Fyn and Yes, derived by mutagenesis of their C-terminal inhibitory tyrosine phosphorylation sites to phenylalanine. Wnt5 and active RhoAV12 were described previously [17]. The NS- and LS-Shp2 RNAs contained their respective mutations, D61G, T73I, A462T, or G465A. Phenotypes were assessed at the indicated stages.

### Cell tracing.

Embryos were (co-) injected at the one-cell stage with 0.25% 4,5-dimethoxy-2-nitrobenzyl (DMNB)-caged fluorescein dextran (10,000 MW, Molecular Probes). Uncaging was done as described [17] at shield stage (6 hpf) using an Axioplan microscope, equipped with a UV light source, adjustable pinhole, and 40× objective. Pictures were taken immediately following uncaging, at 80% epiboly (8 hpf) and tailbud stage (10 hpf). The angles for dorsal convergence and anterior extension were determined using NIH imaging software.

### Phosphatase assays.

Purified GST-fusion proteins were directly incubated in PTP assay buffer (20 mM MES buffer pH 6.0, 1 mM EDTA, 150 mM NaCl, 1 mM dithiotreitol, and 10 mM p-nitrophenylphosphate) for 45 min at 30 °C. The reactions were quenched with 0.4 M NaOH, and optical density was measured with a spectrophotometer at 415 nm (wavelength).

## Supporting Information

Figure S1 Morphometry of the Hammerhead Phenotype The distance between the eyes and the distance from the middle of the eyes to the tip of the nose (as indicated in the left panel) was determined in 4 dpf embryos after staining with alcian blue (see Materials and Methods). The ratio of the distance to the tip of the nose and the distance between the eyes was determined and is plotted in the bar graph, depicted in the panel on the right. Four WT and ten Shp2-MO injected embryos were analyzed. There is a significant difference in ratios between the WT (1.87 ± 0.03) and Shp2 knockdown (1.40 ± 0.20), indicating that Shp2 knockdown indeed induced a hammerhead phenotype.
(323 KB PPT)

Figure S2 Shp2 and Wnt5 Knockdown Acts Synergistically to Induce a Hammerhead Phenotype at 4 dpf Shp2-MO and Wnt5-MO were injected at 50% of their normal concentration. By themselves, these injections did not induce defects (compare to morphology at 4 dpf with WT, noninjected control). Coinjection of Shp2-MO and Wnt5-MO did induce the hammerhead phenotype at 4 dpf. Note the blunted face and the increased distance between the eyes in the double-injected embryo (Shp2 + Wnt5).
(583 KB PPT)

Figure S3 Expression of NS- and LS-Shp2 Did Not Induce Defects in Cell Specification Synthetic RNA encoding NS-Shp2 (D61G or T73I) or LS-Shp2 (A462T or G465A) was injected into one-cell stage zebrafish embryos. The embryos were fixed at 6 hpf and in situ hybridization was done with the indicated probes. Animal pole views are depicted here. Expression of the mesendodermal marker *ntl* and of the organiser marker *gsc* remained unchanged in the NS- and LS-Shp2–expressing embryos.
(208 KB PPT)

### Accession Numbers

The National Center for Biotechnology Information (NCBI) Entrez database (http://www.ncbi.nlm.nih.gov/sites/gquery?itool=toolbar) accession numbers of the genes and proteins used in this study are: Fyn (Danio rerio), EMBL AJ620748; RhoA (Homo sapiens), swissprot P61586; Shp2 (Danio rerio), NCBI NM_199846; Shp2 (Homo sapiens), swissprot Q06124; Wnt5a (Mus musculus), swissprot P22725; and Yes (Danio rerio), EMBL AJ620749.

Identifiers of the human Noonan and LEOPARD syndromes are OMIM 163950 and OMIM 151100, respectively.

## Abbreviations

CE: convergence and extension
dpf: d post fertilization
GFP: green fluorescent protein
hpf: h post fertilization
LS: LEOPARD syndrome
MO: morpholino
NS: Noonan Syndrome
PTP: protein-tyrosine phosphatase
SFK: Src family kinase
SH2: Src homology 2
WT: wild type
